# Exploring Bioinformatics Tools to Analyze the Role of CDC6 in the Progression of Polycystic Ovary Syndrome to Endometrial Cancer by Promoting Immune Infiltration

**DOI:** 10.3390/ijms252312974

**Published:** 2024-12-03

**Authors:** Yuhang Song, Jing Zhang, Yao Li, Lufeng Cheng, Hua Song, Yuhang Zhang, Guoqing Du, Sunyue Yu, Yizhou Zou, Qi Xu

**Affiliations:** 1 School of Basic Medicine, Xinjiang Medical University, Urumqi 830054, China; syh6542@126.com (Y.S.); yaoli@stu.xjmu.edu.cn (Y.L.); guoqing_du1001@126.com (G.D.); 2 School of Clinical Medicine, Xinjiang Medical University, Urumqi 830054, China; 13565834658@163.com (H.S.); 15293925014@163.com (Y.Z.); 15199033749@163.com (S.Y.); 3 Department of Immunology, School of Basic Medicine, Central South University, Changsha 410017, China; 226501024@csu.edu.cn; 4 Basic Medical College, Xinjiang Medical University, Urumqi 830054, China; lfcheng@xjmu.edu.cn

**Keywords:** CDC6, polycystic ovarian syndrome, endometrial cancer, immune infiltration

## Abstract

Cell division cycle 6 (CDC6) is essential for the initiation of DNA replication in eukaryotic cells and contributes to the development of various human tumors. Polycystic ovarian syndrome (PCOS) is a reproductive endocrine disease in women of childbearing age, with a significant risk of endometrial cancer (EC). However, the role of CDC6 in the progression of PCOS to EC is unclear. Therefore, we examined CDC6 expression in patients with PCOS and EC. We evaluated the relationship between CDC6 expression and its prognostic value, potential biological functions, and immune infiltrates in patients with EC. In vitro analyses were performed to investigate the effects of CDC6 knockdown on EC proliferation, migration, invasion, and apoptosis. CDC6 expression was significantly upregulated in patients with PCOS and EC. Moreover, this protein caused EC by promoting the aberrant infiltration of macrophages into the immune microenvironment in patients with PCOS. A functional enrichment analysis revealed that CDC6 exerted its pro-cancer and pro-immune cell infiltration functions via the PI3K-AKT pathway. Moreover, it promoted EC proliferation, migration, and invasion but inhibited apoptosis. This protein significantly reduced EC survival when mutated. These findings demonstrate that CDC6 regulates the progression of PCOS to EC and promotes immune infiltration.

## 1. Introduction

Polycystic ovarian syndrome (PCOS) is a common reproductive endocrine disease that affects 4–10% of women of childbearing age worldwide. Its clinical symptoms include chronic anovulation, ultrasound visibility of polycystic ovary morphology, and hyperandrogenism [1]. PCOS is characterized by high heterogeneity, complex symptoms, and difficult treatment. The consequent endocrine and metabolic abnormalities can also lead to various complications and increase the risk of endometrial cancer (EC) [2]. Women with PCOS are 3–4 times more likely to develop endometrial cancer than healthy women [3].

EC is the sixth most common malignant tumor in women, and its incidence and mortality continue to increase annually. It is estimated that the prevalence of this disease will increase by 50–100% by 2030 [4,5]. The primary clinical symptoms of EC include dysfunctional uterine bleeding, menorrhagia, menstrual irregularities, and infertility [6]. PCOS is a major risk factor for the development and progression of type I EC [7,8]. Elevated estrogen levels, hyperinsulinemia, and decreased apoptosis play key roles in the occurrence [9,10]. However, the exact underlying molecular mechanism remains unclear. PCOS serum-derived exosomal miR-27a-5p stimulates EC cell migration and invasion [11]. Matà et al. [12] reported that overexpression of the PI3K/Akt signaling pathway underlies the development of endometrial hyperplasia and carcinogenesis in patients with PCOS characterized by insulin resistance (PCOS-IR). In addition, IGF1/IGFBP pathway-related genes may contribute to an increased risk of EC in women with PCOS [13]. However, the molecular mechanism underlying EC in patients with PCOS remains unclear. This has hindered effective clinical prevention and treatment.

Cell division cycle 6 (CDC6), sited on human chromosome 17q21.3, is a vital regulatory protein for the beginning of DNA copying in eukaryotic cells. It mainly binds to the initiation recognition complex and encourages the transition of the cell cycle from G1 to S phase [14]. This protein plays a central role in various human malignancies. For example, CDC6 expression is markedly increased in breast cancer, particularly estrogen receptor (ER)-negative breast cancer. This suggests that it is a potential prognostic marker and therapeutic target for breast cancer [15]. In prostate cancer, CDC6 exerts its carcinogenic effects by enhancing PIK3-AKT signaling [16]. Similarly, changes in PI3K signaling and partial inhibition of AKT phosphorylation are highly correlated with a reduction in CDC6 levels [17]. In addition, overexpression of CDC6 in non-neoplastic mouse epithelial cells leads to malignant transformation with epithelial–mesenchymal transition characteristics [18,19,20]. However, its role in the progression of PCOS to EC remains unclear.

The infiltration of immune cells and their interaction with cancer cells form a unique tumor immune microenvironment (TIME) [21]. In the tumor immune response, the type of immune cell infiltration is crucial for the prognosis of tumor patients, because it determines whether the type of immune response tends to be antitumor or protumor [22]. The fraction of immune cell infiltration plays crucial roles in tumor growth, metastasis, and therapeutic resistance [23]. Immune cell infiltration (ICI) in the TIME plays a decisive role in patient prognosis. Many studies have shown that there is significant ICI in uterine corpus endometrial carcinoma (UCEC) tissues, and these immune cells are involved in tumor development [24]. Recent research has suggested that many genes and proteins have impacted the regulation of immune infiltration; for example, the expression of RILPL2 may regulate immune infiltration in the immune microenvironment of EC specimens, thus directly and/or indirectly regulating immune monitor and influencing the progression of the tumor [25]. Wang et al. found that PHF6 KD endometrial carcinoma cells could promote the infiltration of T cells, which further indicated that PHF6 played an essential role in the tumor immune microenvironment in UCEC patients [26]. However, the role of CDC6 in the progression of PCOS to EC is unclear. CDC6 can also promote the formation of an immune environment in the development process of some cancers. In the study conducted by Pu et al. [27], the prognostic relevance and immunological significance of CDC6 across a wide spectrum of cancers was underscored. Notable associations were observed between CDC6 expression and the tumor mutational burden (TMB) and microsatellite instability (MSI), as well as immune cell infiltration. As for clear cell renal cell carcinoma (ccRCC), CDC6 was also related to the TMB, immune checkpoint molecules, tumor microenvironment, and immune infiltration [28]. At the same time, CDC6 was overexpressed in HCC tissues and was negatively associated with the overall and disease-free survival in HCC patients and was related to immune cell infiltration in HCC microenvironments [29]. Consequently, we propose that CDC6 may play a pivotal role in promoting immune infiltration during the progression from PCOS to endometrial cancer, which not only has the potential to elucidate a significant connection between these two gynecological disorders but also offers new theoretical insights for immune-targeted therapies.

## 2. Results

### 2.1. Abnormal Infiltration of Immune Cells Promotes Carcinogenesis in PCOS

**Figure 1:**
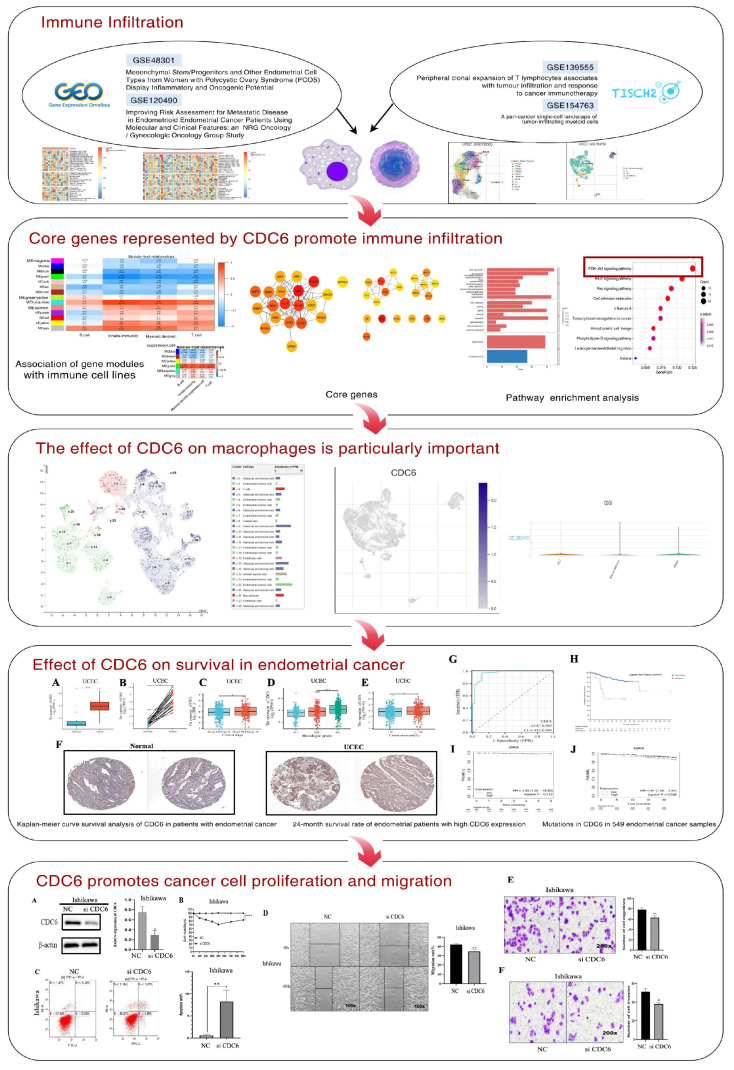
Illustration of the research flow chart.

**Figure 2:**
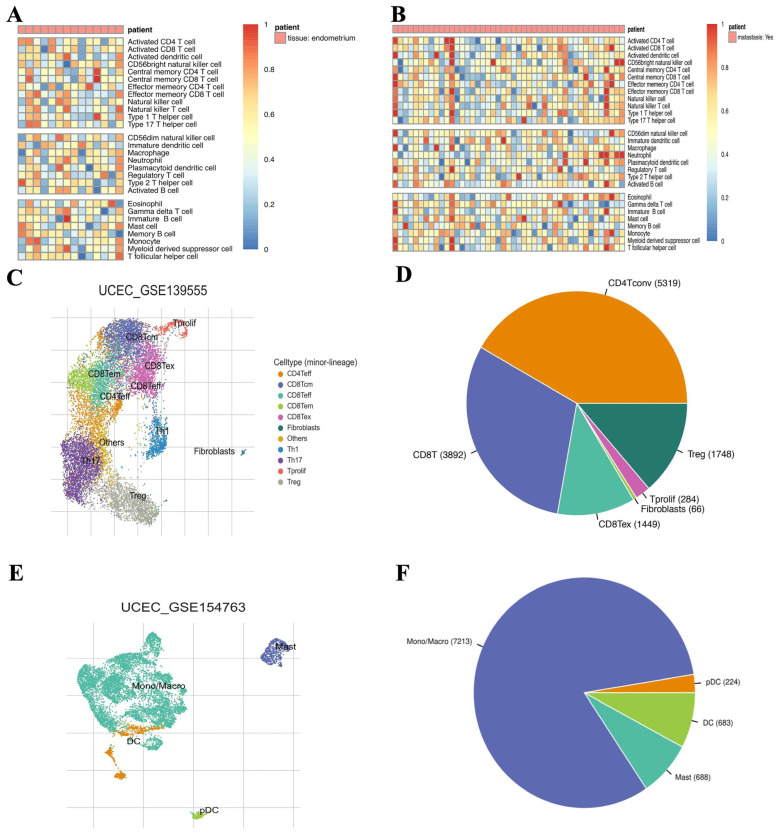
Abnormal invasion of immune cells promotes the occurrence of PCOS carcinogenesis. (**A**) Heatmap of immune cell subsets in PCOS samples with a tendency to become cancerous. (**B**) Heatmap of immune cell subsets in an endometrial cancer sample. The expression (**C**) and expression ratio (**D**) of each immune cell subset in the GSE139555 dataset, as analyzed based on the TISCH database. The expression (**E**) and expression ratio (**F**) of each immune cell in the GSE154763 dataset, as analyzed based on the TISCH database.

This study intended to reveal the association between CDC6 expression and endometrial carcinogenesis during PCOS (Figure 1). we performed a single-cell immune-infiltration analysis using an ssGSEA of carcinomatous PCOS and EC samples to determine the effect of immune cell infiltration on carcinogenesis in patients with PCOS. The infiltration abundances of central memory T cells, monocytes, immature B cells, and macrophages were significantly increased in PCOS with a tendency toward cancer. In contrast, the infiltration abundances of neutrophils and NK cells were relatively low (Figure 2A).

The infiltration abundance of activated T cells, macrophages, and neutrophils was significantly increased, whereas that of monocytes and effector memory T cells was relatively decreased in endometrial carcinoma (Figure 2B). These results indicated an abnormally high expression of immune cells in PCOS and EC. This suggests that the abnormal infiltration of immune cells might play an important role in the carcinogenesis of PCOS, and in the occurrence and progression of EC. Macrophages were significantly upregulated in both the PCOS and EC groups, implying an important effect.

We further verified the association between immune cell infiltration and endometrial carcinoma using the TISCH 2 database. Although various immune cells were labeled in the GSE139555 cohort (Figure 2C), the most significant was the T cell line (Figure 2D), which comprised CD4^+^ and CD8^+^T cells. In contrast, macrophages were significantly labeled (Figure 2F) in the GSE154763 cohort (Figure 2E). In summary, the infiltration of immune cells is closely correlated to the carcinogenesis of PCOS and EC. In particular, the role of macrophages and T cells is more significant, indicating that they may be the most important cells in the carcinogenic role of immune infiltration.

### 2.2. CDC6 Promotes the Formation of the TME

**Figure 3:**
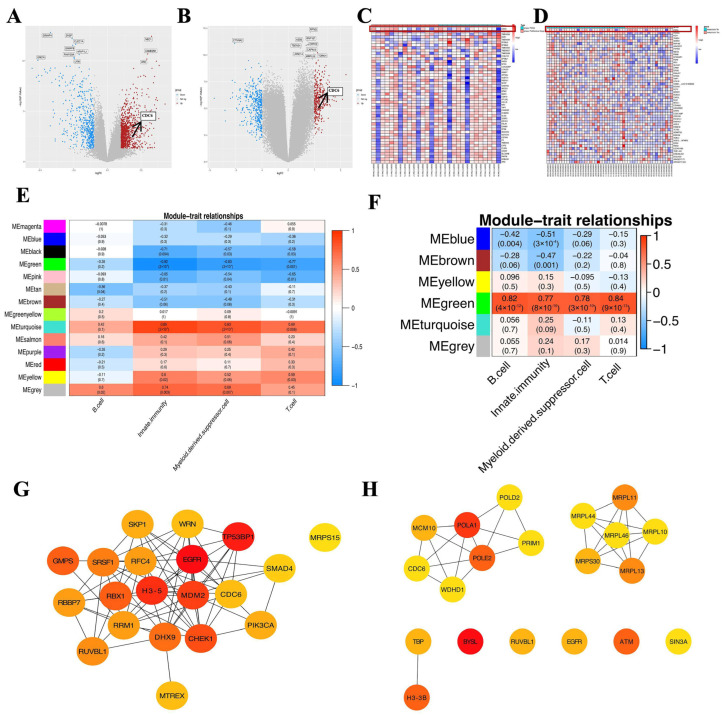
CDC6 promotes the formation of an immune tumor microenvironment. (**A**) Volcanogram of DEGs between normal and PCOS endometrial tissues. (**B**) Volcanogram of DEGs between EC breast and non-breast metastases. (**C**) Heatmap of the top 50 upregulated DEGs between normal and PCOS endometrial tissue. (**D**) Heat map of the top 50 upregulated DEGs between EC breast and non-breast metastases. (**E**) Heatmap of the correlation between module-characteristic genes and immune cells in a PCOS sample. The black, green, pink, sky blue, and yellow modules indicate high association with innate immunity and T cells. The rows represent module-characteristic genes (MEs) and their colors, whereas the columns represent immune cells. Red indicates a positive correlation between the module gene and immune cell. Blue indicates a negative correlation. The darker the color, the greater the relevance. The correlation coefficient and *p*-value are displayed in each cell. (**F**) Heatmap of the correlation between module-characteristic genes and immune cells in EC samples. The green, brown, and blue modules indicate high correlation with the innate immune system. (**G**) The top 20 genes with the degree value of the central module in the PCOS sample. (**H**) The top 20 genes in the degree value of the central module in the EC sample.

We first identified the expression levels of the DEGs to identify the genes related to immune cell infiltration. In total, 978 and 432 genes with upregulated and downregulated expression, respectively, were identified in the PCOS group (Figure 3A). In EC, 334 and 309 genes with downregulated and upregulated expression, respectively, were identified (Figure 3B). A heat map was utilized to illustrate the differential expression of the top 50 genes with upregulated expression, including *CDC6*. The expression of *CDC6* was significantly upregulated in both datasets. Overall, *CDC6* expression was dysregulated in both PCOS and EC (Figure 3C,D). Therefore, CDC6 may be correlated with the abnormal infiltration of immune cells.

We aimed to elucidate the effect of CDC6 expression on ssGSEA-targeted immune cell infiltration. We constructed an expression relationship between the immune cells and PCOS and EC samples to obtain clinical data for a WGCNA (Supplementary Tables S1 and S2). The black, green, pink, sky blue, and yellow modules in PCOS were highly correlated with innate immunity and the T cell line. In contrast, the B cell line was not significantly correlated with any module (Figure 3E). Next, we performed the same analysis on the EC samples. The green segment showed a high connection to T, B, and innate immune cells, whereas the brown and blue modules showed a high correlation with innate immune cells (Figure 3F). Therefore, we selected the innate immune cells and the corresponding green, brown, and blue modules as the central modules for the subsequent analyses.

A PPI network of the central modular genes was created to clarify the representation of *CDC6* in the modular genes. *CDC6* occupied an important position among the first 20 genes of degree value screened using the cytoHubba plug-in (Figure 3G,H). Therefore, our results showed that CDC6 has an impact on driving the abnormal infiltration of immune cells.

### 2.3. CDC6 Is Abnormally Highly Expressed in PCOS Carcinomatosis and EC and Plays a Carcinogenic Role Through the PI3K-AKT Pathway

**Figure 4:**
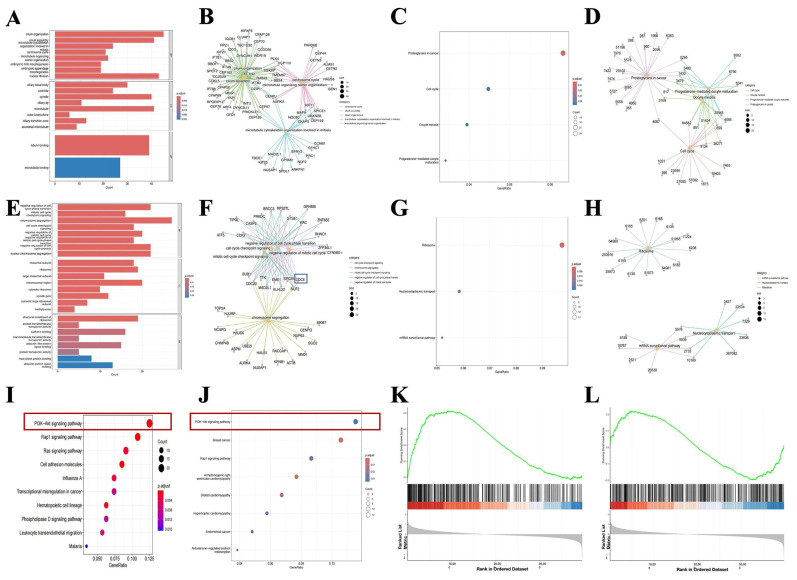
CDC6 is abnormally overexpressed in PCOS carcinogenesis and EC and exerts oncogenic effects through the PI3K-AKT pathway. (**A**) GO enrichment analysis of CDC6-related DEGs in PCOS samples with a tendency to be carcinogenic. (**B**) Correlation network diagram of CDC6-related DEGs with GO enrichment functional pathways in PCOS samples with carcinogenic potential. (**C**) Significant enrichment bubble map of the KEGG pathway of CDC6-related DEGs in PCOS samples with a tendency to be carcinogenic. (**D**) Correlation network diagram of CDC6-related DEGs with KEGG enrichment functional pathway in PCOS samples with carcinogenic potential. (**E**) GO pathway enrichment bar graph of CDC6-related DEGs in EC samples. (**F**) Diagram of the association network between CDC6-related DEGs and GO-enriched functional pathways in EC samples. (**G**) Significant enrichment bubble map of the KEGG pathway for CDC6-related DEGs in EC samples. (**H**) Correlation network diagram of CDC6-related DEGs with KEGG-enriched functional pathways in EC samples. (**I**) Downregulated DEG pathway enrichment bubble map in PCOS samples with carcinogenesis potential. Of these, the PI3K-AKT pathway was significantly enriched. (**J**) Downregulated DEG pathway enrichment bubble map in EC samples; the PI3K-AKT pathway was significantly enriched. (**K**) Gene set enrichment analysis of DEGs upregulated in PCOS samples with a tendency to be carcinogenic. (**L**) Gene set enrichment analysis of DEGs in PCOS samples with carcinogenic potential.

We performed a functional enrichment analysis to further investigate the biological role of CDC6 in PCOS and EC. As shown in Figure 4A–D, the DEGs related to CDC6 were mostly enriched in cell division and movement in PCOS with a carcinomatous tendency. The expression of these genes and that of *CDC6* was simultaneously upregulated. In addition, these genes regulated cell proliferation, apoptosis, and tumor invasion (Supplementary Tables S3 and S4). Endometrial carcinomas were mainly enriched in cell division-related organelles and cell transporters (Figure 4E–H). This suggests that CDC6 might provide better conditions for cancer cell growth and promote metastasis and deterioration (Supplementary Tables S5 and S6).

The analyses of the pathway enrichment of the genes with downregulated expression revealed that the PI3K-AKT pathway was meaningfully enriched in both PCOS and EC (Figure 4I,G). This suggests that the PI3K-AKT pathway may play a vital role in the carcinogenesis of PCOS to endometrial carcinoma. To further clarify the relationship between this pathway and DEG expression, we performed a GSEA. Although the PI3K-AKT pathway was not significantly enriched in the KEGG analysis of genes with upregulated expression, it showed a strong correlation with these genes. In addition, it showed a significant bidirectional enrichment tendency with respect to all genes (Figure 4K,L).

In summary, CDC6 is highly carcinogenic, and its effects may be mediated by the PI3K-Akt pathway. Therefore, high CDC6 expression can lead to a carcinomatous tendency in PCOS and further carcinogenesis into EC. Furthermore, it can affect the metastasis of malignancy.

### 2.4. CDC6 Causes the Progression of PCOS to EC Through Abnormal Infiltration of Macrophages

**Figure 5:**
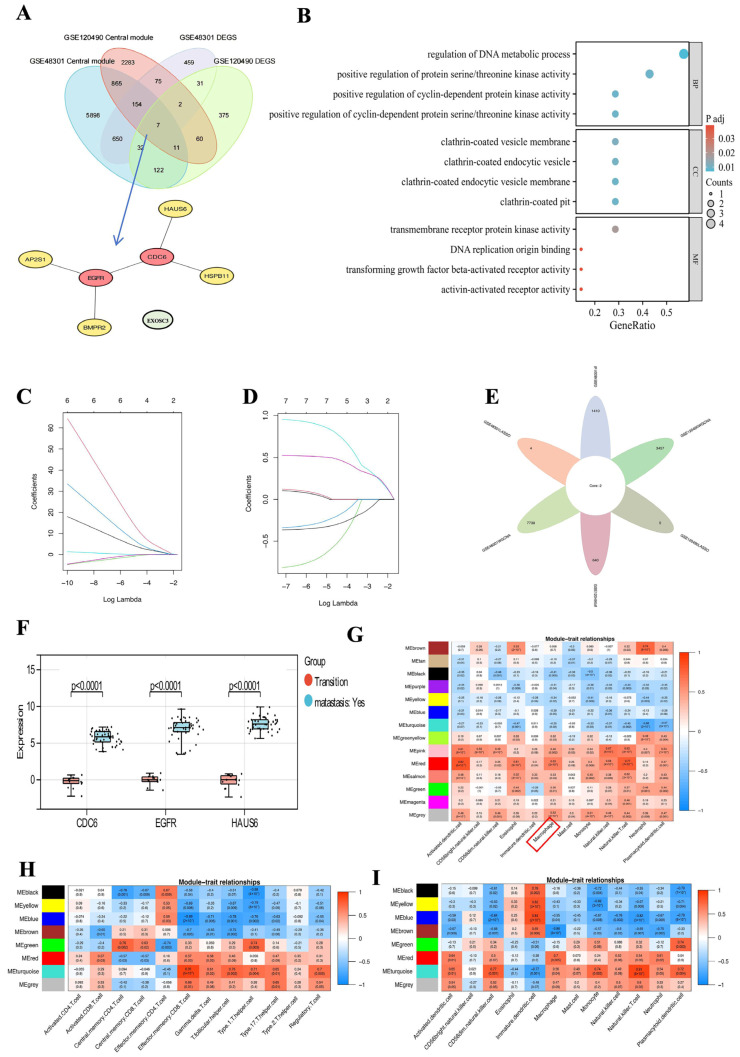
CDC6 causes the progression of PCOS to EC through abnormal macrophage invasion. (**A**) Venn diagram of the intersection of DEGs between normal and PCOS endometrial tissue, DEGs between EC breast and non-breast metastasis samples, central module genes in PCOS samples, and central module genes in EC samples. PPI network diagram of seven candidate genes, with *CDC6*/*EGFR* at the core. (**B**) GO and KEGG enrichment analyses of the seven genes. (**C**) Variable coefficient change characteristic plot of the PCOS sample with a carcinogenic tendency, minimum absolute contraction = 0. The value of the dataset as a biomarker was analyzed. (**D**) Variation characteristics of the variable coefficient of the EC samples, with minimum absolute contraction. (**E**) The genes in the PCOS sample dataset with a carcinogenic tendency after LASSO analysis, the Venn diagram of the genes after LASSO analysis, and the seven candidate genes in the EC sample dataset. The identification genes *CDC6* and *HAUS6* were obtained. (**F**) Expression and diagnostic values of *CDC6*, *EGFR*, and *HAUS6* in PCOS samples with a tendency to become cancerous. (**G**) Heatmap of the correlation between innate immune subset module genes and immune cell invasion in the EC sample dataset. WGCNA of module–feature relationships between innate immune subsets and immune cell infiltration. The red and pink modules associated with the innate immune subset have the highest correlation coefficients and were identified as the core modules in the EC sample dataset. (**H**) Heatmap of the correlation between T cell subset module genes and immune cell infiltration in PCOS samples with a tendency to become cancerous. (**I**) Heatmap of the correlation between innate immune subset module genes and immune cell infiltration in PCOS samples with a carcinogenic tendency.

We overlapped the screened DEGs with the central modules of the innate immune and T cells obtained after the WGCNA to clarify whether the association of CDC6 between PCOS and EC was achieved through the infiltration of immune cells. Seven candidate genes were obtained: *HSPB11*, *AP2S1*, *BMPR2*, HAUS augmin-like complex subunit 6 (*HAUS6*), *EXOSC3*, *CDC6*, and epidermal growth factor receptor (*EGFR*) (Figure 5A). The subsequent construction of PPI networks across these proteins based on the STRING database revealed the important role of CDC6. However, because EXOSC3 was not directly related to other proteins, we evaluated the correlation between the other six proteins (Figure 5A). We performed GO and KEGG enrichment analyses on the six interacting protein-encoding genes, which are mainly involved in the regulation of DNA metabolism (Figure 5B). Our results suggest that CDC6 plays an important role in promoting the abnormal infiltration of immune cells and cancer onset. Moreover, we performed a LASSO regression analysis on the six genes to further evaluate the value of *CDC6* as a biomarker; *CDC6* was retained in both sample groups (Figure 5C,D) (Supplementary Tables S7 and S8). The role of *HAUS6* was also identified (Figure 5E). HAUS6 is one of the eight subunits of the augmin complex required for microtubule (MT)-dependent MT amplification independent of centrosomes and chromatin during cell division. Moreover, EGFR is an important signaling pathway integration factor for G protein-coupled receptors, steroid receptors, and tyrosine kinases. EGFR plays a major role in many cellular processes. Additionally, it is important for the production of reactive oxygen species and release of macrophage inflammatory mediators. These results suggest that CDC6 may be related to EGFR and HAUS6, potential biomarkers that lead to the excessive infiltration of PCOS immune cells and, thus, the carcinogenesis of PCOS. Therefore, we further determined the expression levels of *CDC6*, *EGFR*, and *HAUS6* during PCOS carcinomatosis to endometrial carcinoma. The expression levels of these genes increased with the development of cancer, owing to their higher expression in the metastasis group than in the transition cancer group (Figure 5F).

The WGCNA of individual immunocytes revealed that CDC6 facilitates the progression of PCOS to EC by affecting macrophages. Of the innate immune cells involved in PCOS and EC, macrophages exhibited a significantly higher association with gene modules represented by *CDC6*. This suggests that macrophages have enhanced sensitivity to gene regulation and are more influenced by *CDC6*, *EGFR*, and other genes compared with other immune cells (Figure 5G–I). Therefore, macrophages and monocytes may play important roles in the onset and development of PCOS and EC. In particular, the central genes represented by *CDC6* have a significant effect on macrophages. In addition to its carcinogenic effects, this gene most likely affects the immune microenvironment and occurrence of carcinomatosis by acting on macrophages.

### 2.5. Single-Cell Analysis of CDC6 in Endometrial and Endometrial Carcinoma Tissues

**Figure 6:**
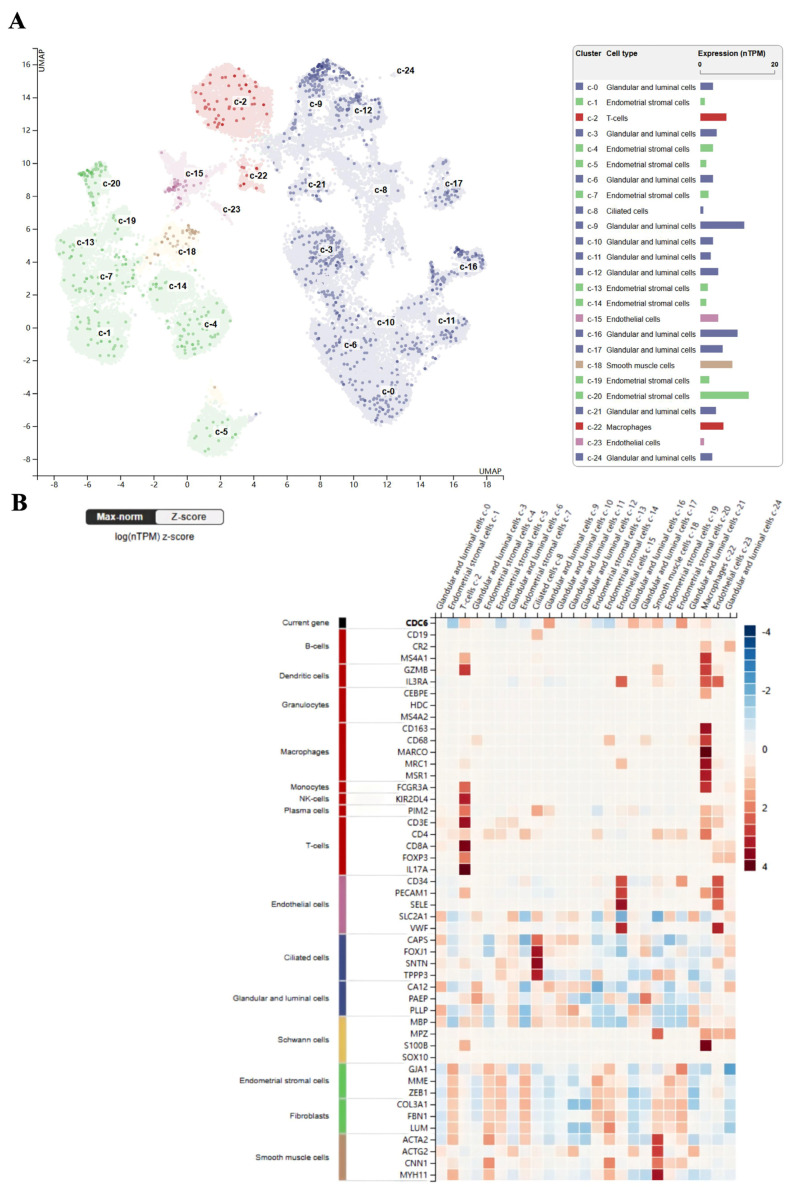
The infiltration of endometrial immune cells, as analyzed through single-cell sequencing in the HPA database. (**A**) Types and distribution of immune cells in the endometrium. (**B**) Heatmap of the correlation between CDC6 and immune cell marker genes in the endometrium.

**Figure 7:**
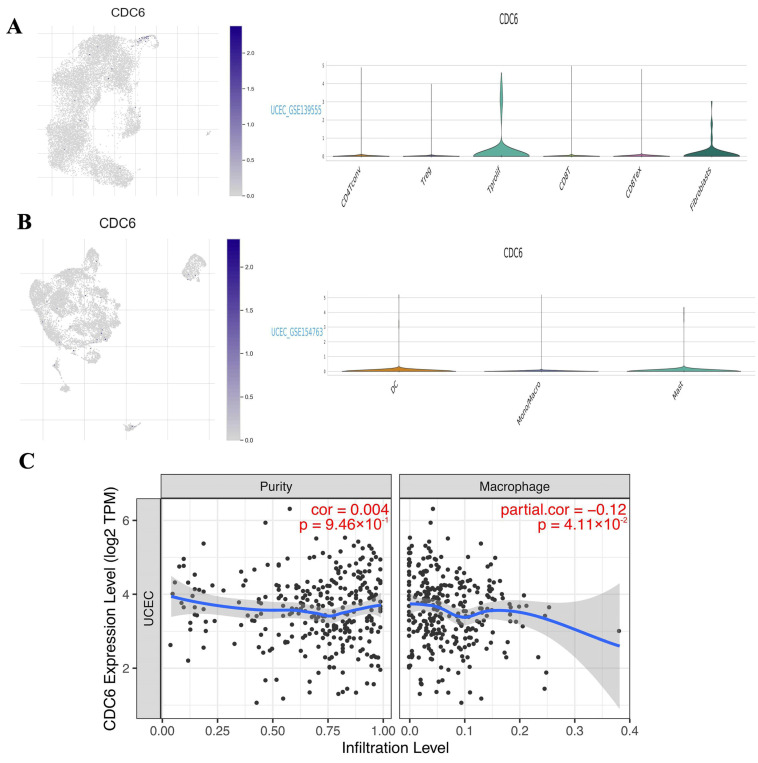
CDC6 is significantly expressed in macrophages of EC. (**A**) and (**B**) show CDC6 expression profiles in the single-cell GSE139555 and GSE154763 datasets; (**C**) CDC6 expression in patients with EC in the TIMER data was significantly correlated with macrophage immune infiltration.

The analysis of single-cell endometrial tissue data from the HPA database revealed the expression of CDC6 in 24 cell types. Of these, the immune-infiltrated cells were mainly T cells and macrophages (Figure 6A). CDC6 expression was positively correlated with T cells and macrophages (Figure 6B). The TISCH2 database revealed the expression of CDC6 in CD4^+^T, Treg, Tprolif, CD8^+^T, and CD8 Tex cells, and in fibroblasts in the single-cell data of EC tissues (GSE139555) (Figure 7A). Moreover, CDC6 was expressed in DC, macrophages, monocytes, and mast cells in EC tissues (GSE154763) (Figure 7B). These findings indicate that innate immune cells were highly correlated with both PCOS and EC. Macrophages play a significant role in innate immunity. Therefore, we verified the relationship between macrophages and CDC6 using the TIMER database. The results are presented in the figures. Although the trend in the samples is slightly discrete, the important role of marker genes in macrophage infiltration was clear (Figure 7C). In summary, CDC6 expression is closely related to the infiltration of the TME, suggesting that CDC6 may affect the immune response in endometrial carcinoma.

### 2.6. CDC6 Expression Levels in Endometrial Carcinoma Were Correlated with Clinical Parameters, Diagnostic Value, and Prognostic Value

**Figure 8:**
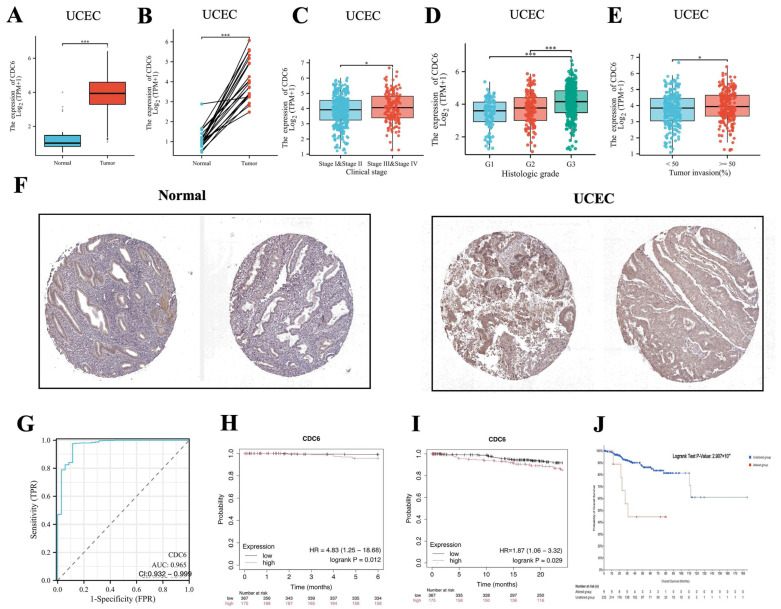
(**A**) mRNA expression levels of CDC6 in EC and normal tissues, as determined based on TCGA database. (**B**) mRNA expression levels of CDC6 in EC and paired adjacent tissues, as determined using TCGA database. The correlation between CDC6 expression and the pathological stages of UCEC (**C**), the histologic G grades in UCEC (**D**), and tumor invasion in UCEC (**E**). (**F**) The protein levels of CDC6 in normal and UCEC tissues, as determined based on the GEPIA database. (**G**) Receiver operating characteristic (ROC) curve for CDC6 expression in UCEC. (**H**,**I**) Kaplan–Meier survival curves showing that elevated CDC6 expression is associated with a worse survival (6 and 24 months) in UCEC. (**J**) The overall survival time related to CDC6 mutation was statistically poorer.(* *p* < 0.05, *** *p* < 0.001).

The expression of CDC6 in UCEC was higher than that in the corresponding control and adjacent normal tissues (Figure 8A,B). Therefore, we investigated the association between CDC6 expression and pathological characteristics. CDC6 had a higher affinity for UCEC in the advanced and G stages (Figure 8C,D). In addition, CDC6 expression in UCEC was higher in patients with tumor invasion (≥50%) (Figure 8E). The HPA database revealed high CDC6 expression levels in UCEC (Figure 8F). A receiver operating characteristic (ROC) curve analysis was used to assess the diagnostic value of CDC6 for UCEC. CDC6 had a high accuracy (AUC = 0.965, CI: 0.932–0.999) in predicting UCEC (Figure 8G). To further explore the impact of changes in CDC6 expression on the overall survival (OS) rate of patients with UCEC, we performed a log-rank test on *CDC6* in the cBioPortal for the cancer genomics database; the results are expressed as Kaplan–Meier curves. CDC6 had a significant effect on the survival of patients with EC (Figure 8H,I). A Kaplan–Meier plotter was used to clarify the impact of abnormal CDC6 expression on the survival rate of patients with EC. As shown in Figure 8H,I, the 6- and 24-month survival rates of the patients with EC characterized by high CDC6 expression were significantly lower than those of the low-expression group (*p* < 0.05). The red line indicates the survival rate of patients with *CDC6* mutation, whereas the blue line indicates the survival rate of patients with no mutation in the given gene. The survival difference related to *CDC6* mutation was statistically significant (*p* = 2.907 × 10^−4^) (Figure 8J). In summary, abnormal CDC6 expression plays an important role in the occurrence of endometrial carcinoma. Similarly, CDC6 mutation is an important marker of survival and prognosis in patients with EC.

### 2.7. Variation in CDC6 Mutation in UCEC

**Figure 9:**
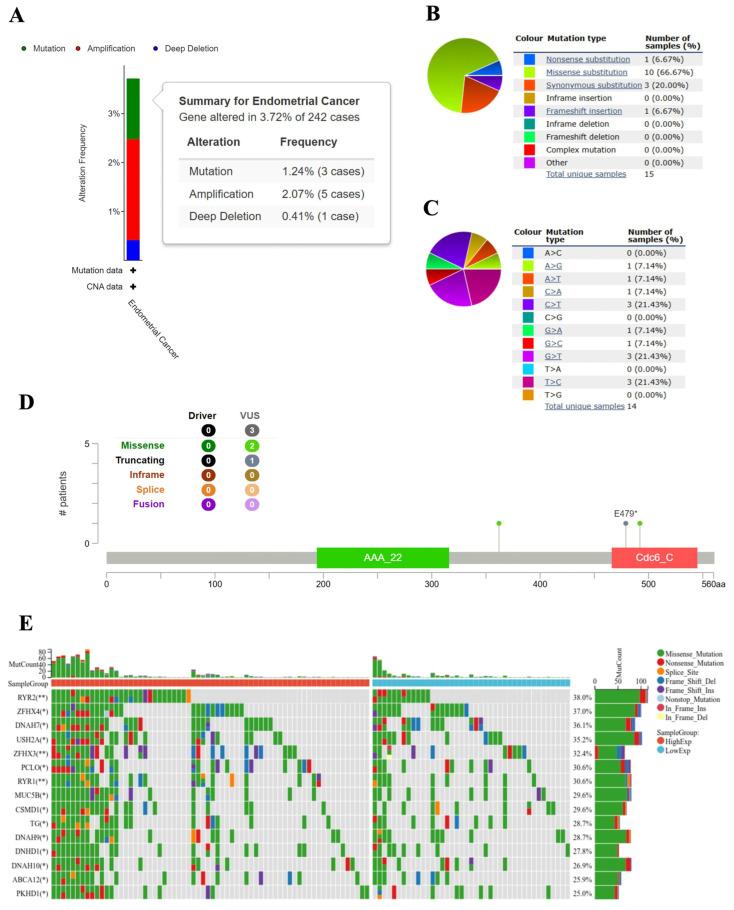
(**A**) Variation types and frequency of CDC6 in UCEC, as determined based on the cBioPortal database. (**B**) and (**C**) show the mutation types of CDC6 in UCEC, as determined based on the COSMIC database. (**D**) Mutation sites are shown. (**E**) The Oncoplot of the somatic mutant landscape in high- and low-CDC6 expression groups in UCEC (* *p* < 0.05, ** *p* < 0.01).

The cBioPortal database revealed that 3.72% (9/242) of the patients with UCEC had variations in CDC6: mutations in 1.24%, amplifications in 2.07%, and deep deletions in 0.41% (Figure 9A). Therefore, the COSMIC database was used to further evaluate the mutation types. Missense substitutions, nonsense substitutions, synonymous substitutions, and frameshift insertions occurred in 66.67%, 6.67%, 20%, and 6.67% of the UCEC samples, respectively. Furthermore, the base substitutions were mainly A>G (7.14%), A>T (7.14%), C>A (7.14%), C>T (21.43%), G>A (7.14%), G>C (7.14%), G>T (21.43%), and T>C (21.43%) (Figure 9B,C). The types, sites, number of CDC6 cases, and the genetic alterations are shown in Figure 9D. A missense mutation in CDC6 was the main type of genetic alteration. We further found an E479* alteration in the CDC6_C domain, which was detected in one case of CDC6. Finally, CDC6 truncation was observed (Figure 9D).

To determine whether the CDC6 expression levels were associated with specific genomic characteristics in UCEC, we performed a somatic mutation analysis based on the CDC6 expression levels using the “maftools” package in the TCGA-UCEC database. The top 15 mutational genes were displayed. A high frequency of mutations in *RYR2* (38%), *ZFHX4* (37%), and *DNAH7* (36.1%) was observed in the high-CDC6 expression group (Figure 9E).

### 2.8. CDC6 Promotes the Proliferation, Migration and Invasion of Endometrial Carcinoma Cells and Inhibits Apoptosis

**Figure 10:**
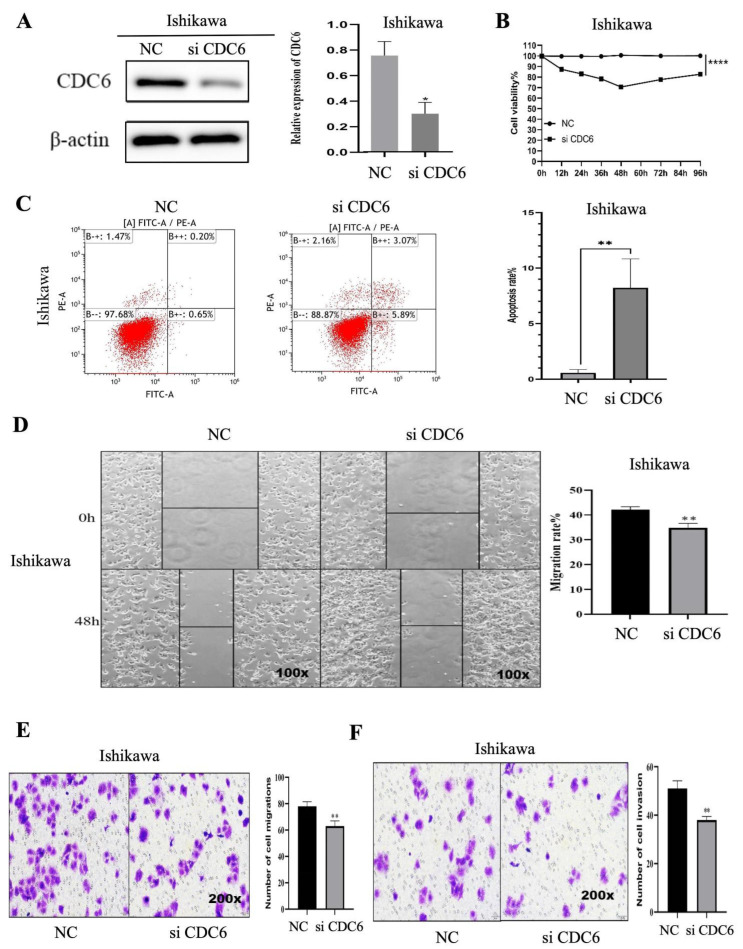
(**A**) Relative protein expression of CDC6 in EC cells determined the efficiency of siRNA, as evaluated using Western blotting. (**B**) CCK8 analysis of the promotion of EC cell proliferation by CDC6. (**C**) Flow cytometric analysis of the silencing of CDC6 to inhibit apoptosis. (**D**) The silencing of CDC6 inhibited the migratory ability of EC cells in the wound healing assay. (**E**,**F**) CDC6 knockdown in EC cells reduced their invasion ability in the Transwell assay (* *p* < 0.05, ** *p* < 0.01, **** *p* < 0.0001).

We evaluated the role of CDC6 in the proliferation, migration, invasion, and apoptosis of EC cells. First, we successfully transfected Ishikawa cells with CDC6 siRNA. The Western blotting results showed that the protein expression level in the siCDC6 group was significantly lower than that in the NC group (Figure 10A). Furthermore, the CCK-8 assay was used to evaluate the effect of CDC6 on EC cell proliferation. The silencing of CDC6 inhibited the proliferation of Ishikawa cells (Figure 10B). Moreover, the flow cytometric analyses revealed that the rate of apoptosis in the siCDC6 group was significantly higher than that in the NC group (Figure 10C). We performed wound healing and Transwell assays to further determine whether CDC6 affects the migration and invasion of EC cells (Figure 10D). The migration and invasion of the siCDC6 group were significantly lower in the NC group than in the control group (Figure 10E,F). These results suggest that CDC6 promotes the migration and invasion of endometrial carcinoma cells but inhibits apoptosis.

## 3. Discussion

CDC6 is a key permissive factor and regulator of DNA replication in eukaryotic cells [30,31,32]. Cellular replication is fraught with danger, precisely regulates the replication of DNA within cells, is essential for overall survival, and ensures the transmission of genetic material from offspring to offspring [33]. This process is partially regulated by replication-permissive factors, of which CDC6 is a key molecule [34,35]. CDC6 is mainly involved in the assembly of the G1 phase “pre-replication complex (pre-RC) [36]” and regulates the G2/M phase checkpoint through Chk1 activation, thereby ensuring normal progression of the cell cycle [37,38]. CDC6 also regulates cell division in the S phase and is a key component in the mitotic CKD-activated cascade during early embryonic division. Its polo is related to Xic1, a true CDK1 inhibitor upstream of Aurora A and polo-like kinase 1, both of which are CKD1 activators. The inclusion of CDC6/Xic1-dependent inhibition in the CKD1 activation cascade explains why cells divide at a specific time, how the pathways involved in the real-time regulation of cell division are integrated, and how mitotic time is fine-tuned [39]. CDC6 plays an important role in multiple stages of cell division, indicating that its abnormal expression may affect this process, leading to cancer. CDC6 is a true proto-oncogene involved in tumor initiation and progression and is overexpressed in many tumor types [40]. The overexpression of this replication-permissive factor has been consistently observed in various studies, providing strong evidence for its significant role in tumor growth and suggesting a broad spectrum of carcinogenic effects [41,42,43]. CDC6 overexpression results in repeated DNA replication. This leads to genomic instability, oncogene activation [44,45], and checkpoint replication through the CDC6-ATR-Chk1 signaling pathway in stages that promote cancer cell survival [46,47].

CDC6 overexpression has been shown in various cancer types. CDC6 is overexpressed in several cancers, such as breast cancer, glioma, renal clear cell carcinoma, ovarian cancer, lung cancer, and chronic myeloid leukemia. Mutations promote the proliferation of breast cancer cells and cell cycle checkpoints to promote cancer cell metastasis [48]. Similarly, the high expression of CDC6 is significantly related to the survival rate and immune infiltration of gliomas. This may occur through the NF-κB signaling, MAPK, and P53 pathways. CDC6 promotes the proliferation, migration, and invasion of glioma cells and inhibits the apoptosis of these cells [49]. CDC6 expression was significantly upregulated in renal clear cell carcinoma tissues compared with adjacent normal renal tissues [28]. In the present study, we identified CDC6 expression as a potential indicator of poor prognosis in patients with renal cell carcinoma [50]. In the present study, we found a high expression of CDC6 in both the PCOS and EC samples with carcinomatous tendencies, which is highly consistent with the expression of CDC6 in various cancers. This suggests that CDC6 is essential for the onset of EC and indicates its correlation with PCOS carcinogenesis.

The TME is a key factor in the proliferation and progression of cancer cells. The TME includes a large number of cells and excessive accumulation of cytokines, chemokines, and growth factors [51,52]. The infiltration of immune cells into the TME can promote the development of cancer cells to a large extent [53,54]. Differences in gene expression during tumor formation may lead to changes in information transmission between immune cells, consequently affecting the activation of immune responses in humans [55]. The expression of *CDC6* has a positive correlation with the tumor purity and the infiltration level range of B cells, CD8^+^T cells, CD4^+^T cells, macrophages, neutrophils, and dendritic cells in hepatocellular carcinoma (HCC) tissues. In addition, the copy number variation of the gene was significantly associated with the infiltration levels of B cells, CD4^+^T cells, macrophages, neutrophils, and dendritic cells. The correlation between the expression of CDC6 and immune cell markers indicated that it plays an important role in the regulation of tumor immunity. Furthermore, CDC6 is related to the infiltration of macrophages and can be used as an independent prognostic factor in patients with renal cell carcinoma [28]. In gliomas, CDC6 expression is positively correlated with macrophage infiltration [49].

Despite the diverse inflammatory components in various cancer types [56], increasing evidence has demonstrated the importance of macrophages in the progression of solid cancers [57]. Macrophages are the key inflammatory effector cells, so better understanding their roles may uncover an effective therapeutic strategy for cancer [58]. In the inflammatory TME, the proportion of macrophages can be as high as 30–50%, and their function has been considered as the ‘soil’ for tumor growth. At the earliest stage of the tumor growth, macrophages polarize to the M1 type to generate an antitumor response. However, once tumors progress past the initial state, the macrophages polarize to M2 to promote tumor progression and malignancy [59,60,61]. M1 macrophages eliminate cancer cells by phagocytosis, antibody-dependent cytotoxicity, vascular damage, and tumor necrosis. M2 macrophages promote tumor growth and progression via enhancing cancer cell survival, angiogenesis, and immune suppression [62,63,64]. Furthermore, the study conducted by Jing et al. revealed that CDC6 primarily influences the infiltration and polarization of proliferating T cells and macrophages [65]. The research conducted by Luo et al. further demonstrates that CDC6 influences immune infiltration within lung adenocarcinoma tissues and exhibits a strong correlation with macrophages [66]. Our study found that the abundance of macrophage infiltration in carcinomatous PCOS and EC samples was abnormally increased. This indicates that macrophages may play an important role in the progression of carcinomatous PCOS to EC, and that the abnormal infiltration of macrophages might be driven by abnormal CDC6 expression.

The PI3K-AKT pathway appeared repeatedly during our analysis. The abnormal regulation of PI3K-AKT signaling is closely related to cancer and autoimmune diseases [67]. In response to activating signals, PI3Ks are recruited to the plasma membrane, where the catalytic subunit dissociates from the regulatory subunit and is activated [68]. The PI3K-AKT pathway can also control most cancer markers, including the cell cycle, motility, survival, metabolism, and genomic instability. In addition, it activates signal transduction through mutations in pathway members [69]. These effects of the PI3K-AKT pathway have been confirmed in patients with gastric adenocarcinoma and melanoma [70,71]. This pathway induces and maintains the expression of cytotoxic T-lymphocyte effector molecules (such as perforin, IFN-γ, and granzyme) and other biomarkers that distinguish memory T cells [72]. PI3K maintains its degranulation activity in NK cells, which contributed to the immunoassay results. This indicates that PI3Ks play a key role in NK cell cytotoxicity [73]. PI3Ks are also involved in the development and maturation of B cells and are important mediators of antigen-receptor signals [74]. This has been reported in studies of prostate cancer [16]. CDC6 regulates the onset of prostate cancer through the PI3K/AKT pathway. It reverses the effect of si-LINC01088 on prostate cancer cells by enhancing PI3K/AKT signaling. In a study on cell cycle arrest caused by plasma membrane damage in human lung epithelial cells, the decrease in CDC6 expression was related to changes in the PI3K signaling pathway and partial inhibition of AKT phosphorylation. Contrastingly, CDC6, which is related to macrophage infiltration, was significantly associated with the PI3K-AKT pathway in carcinomatous PCOS and EC in the present study. This is consistent with the findings of most studies investigating the association between CDC6 and the PI3K-AKT pathway. CDC6 may exert a carcinogenic effect on PCOS through the PI3K-AKT pathway, leading to the eventual development of endometrial carcinoma.

Cancer is a complex disease driven by genetic changes. Missense mutations can cause non-functioning proteins and provide a selective growth advantage for cancer cells. Thus, they serve as the key mechanism for the destruction of important cellular behaviors in cancer, such as cell growth, proliferation, and survival [75,76]. To date, little is known about missense mutations in CDC6 and their effects on survival. The TCGA data analysis revealed that CDC6 is overexpressed in most cancers and is associated with a low survival rate in patients with cancer [77]. However, this result was limited to cancer samples included in the TCGA database and was highly biased. The effect of CDC6 on the survival rate of patients with cancer remains largely understudied. However, a previous study found that the five-year survival rate of the CDC6 high-expression group was lower than that of the low-expression group in colorectal cancer [78]. Furthermore, missense mutation in CDC6 may contribute to the development of non-small cell lung cancer (NSCLC) in non-smoking patients. In addition, the overexpression of CDC6 in NSCLC is associated with a poor prognosis [79,80]. CDC6 overexpression is associated with a low survival rate in many patients with cancer [77]. We reached a similar conclusion in our evaluation of the effect of CDC6 on endometrial carcinoma. The effect on the survival rate of patients with endometrial carcinoma was more significant when a missense mutation of CDC6 occurred than in patients with high CDC6 expression. These results suggest that the abnormal mutation of CDC6 with abnormally high expression compared to non-mutated CDC6 might be a more concerning problem. Therefore, the mutation sites of CDC6 warrant further study. This may provide new theoretical guidance and treatment approaches for the subsequent design of targeted drugs and the extension of patient survival time. We have sufficient evidence to suggest that the abnormal infiltration of macrophages plays an important mediatory role between PCOS and endometrial carcinoma formation. CDC6 promotes the formation of an immune microenvironment in the endometrium and enhances the infiltration of macrophages, which is likely to further facilitate cancer onset via the PI3K-AKT pathway. Although the possible role of the PI3K-AKT pathway remains largely understudied, our findings provide theoretical support to indicate the occurrence of related cancers via this pathway. CDC6 mutation had a significant impact on the survival rate of patients with EC, surpassing the effect seen in cases where CDC6 was only highly expressed. Therefore, CDC6 mutation requires special attention in the study of the survival and prognosis of patients with EC. These findings provide an accurate basis for future follow-up research geared toward the development of effective treatment for patients with EC.

In conclusion, in PCOS and EC sample databases, CDC6 can be screened for since it is highly expressed in endometrial carcinoma and may play an important role in the progression of PCOS to EC. It may regulate macrophages in the TME via the PI3K-AKT pathway to promote endometrial carcinoma formation. In vitro experiments have also shown that CDC6 promotes the proliferation, migration, and invasion of EC cells. This implies that its mutation can affect the overall survival of patients with EC. Therefore, the role of CDC6 in the progression of polycystic ovary syndrome to endometrial cancer by promoting immune infiltration may serve as a new tumor prognostic biomarker and a potential immunotherapy target.

## 4. Materials and Methods

### 4.1. Data Collection and Processing

Gene expression matrices and clinical data were collected from the NCBI Gene Expression Omnibus database. After screening using the R program, we obtained gene expression matrices and clinical data from the GSE171507 (42 normal endometrial samples and 30 endometrial samples from patients with PCOS), GSE48301 (14 PCOS endometrial samples with carcinogenic potential), and GSE120490 (45 endometrial cancer breast metastasis samples) datasets. Differences in *CDC6* mRNA expression between cancer tissues and normal tissues from The Cancer Genome Atlas (TCGA) datasets were explored.

### 4.2. Gene Differential Expression and Enrichment Analyses

The Wilcoxon signed-rank test was used to identify differentially expressed genes (DEGs) in the GSE120490 and GSE48301 datasets. The cut-off values were *p* < 0.05 and |logFC| ≥ 1. Gene Ontology (GO) and Kyoto Encyclopedia of Genes and Genomes (KEGG) enrichment analyses were performed using the clusterProfiler package in R (version 4.3.1).

### 4.3. Single-Cell Immune Infiltration Assay

The infiltration of three types of immune cell lines and 28 types of immune cells in PCOS and EC samples from the GSE120490 and GSE48301 datasets was assessed using single-sample gene set enrichment analysis (ssGSEA) and the GSVA package39 in the R software (v4.3.1). T, B, and innate immune cell lines were used.

### 4.4. Construction of Protein–Protein Interaction Networks

A protein interaction network (PPI) was created using the STRING database and Cytoscape software (v3.6.1). The backbone network was selected from the hub module based on topological characteristics. The Molecular Complex Detection (MCODE) algorithm was used to detect the central genes in the PPI network.

### 4.5. Weighted Correlation Network Analysis

Weighted correlation network analysis (WGCNA) is a systems biology technique used to designate gene association patterns. It can be used to identify gene sets with highly synergistic changes. We used WGCNA to evaluate the association between gene expression profiles and clinical phenotypes based on the GSE120490 and GSE48301 datasets. After R-script screening, the module-characteristic genes and immune cell subtypes previously analyzed through ssGSEA was evaluated using the Pearson correlation test. The optimal module related to the sample characteristics was identified and selected as the central module.

### 4.6. Identification of Biomarkers

The Least Absolute Shrinkage and Selection Operator (LASSO) algorithm based on the glmnet R package (v4.1-8) was used to select gene signatures. Robust gene signatures were identified using LASSO. Diagnostic values for genetic signatures were assessed using ROC curves.

### 4.7. Bioinformatic Analysis Based on Online Tools

The TIMER database is a comprehensive database that enables the analysis of immune cells in different tumors. This database was used to determine the role of marker genes in immune cell infiltration.

BioPortal (https://www.cbioportal.org (accessed on 3 October 2023)) is an open website for the visualization and analysis of cancer genetic data. cBioPortal was used to screen the expression patterns and possible genetic changes in patients with EC under the influence of the identified TME regulatory genes. In addition, survival analysis of core genes was performed.

KM-plotter (https://kmplot.com/analysis/ accessed on 21 June 2024) is an online survival analysis website that evaluates the impact of genes on cancer survival. The KM-plotter database was used to clarify the impact of target genes on the survival of patients with cancer in different years.

TISCH 2 (http://tisch.comp-genomics.org/home/ (accessed on 15 December 2023)) is a web-based tool for the analysis of TME patterns in various tumors based on scRNA-seq datasets. We used this tool to evaluate the relationship between EC and TME in macrophages. GSE139555 dataset: single-cell RNA-seq and TCR-seq from pretreatment samples of tumors, normal adjacent tissues, and peripheral blood from 14 patients with cancer. GSE154763 dataset: single-cell RNA-seq of 48 patients with multiple human cancer types. GSE171507 dataset: RNA-seq of women with PCOS and healthy controls.

The Human Protein Atlas (HPA) is a database designed to map all human proteins in cells, tissues, and organs using various integrated omics techniques to study gene expression differences at the protein level. Therefore, we used it to investigate the expression of CDC6 in normal and cancerous tissues.

### 4.8. Cell Culture

Ishikawa cells (Shanghai Yunbelt Technology, Ltd., Shanghai, China) were cultured in high-glucose DMEM supplemented with 1% penicillin/streptomycin (Biosharp, China) and 10% fetal bovine serum (Lonsera, Suzhou Shuangjun Biotechnology Co., Ltd., Suzhou, China). The cells were then incubated at 36 °C and 5% CO_2_. The medium was changed every 2 days and passaged when confluence reached about 90%.

### 4.9. Ishikawa Cell Transfection

Ishikawa cells were cultured to the logarithmic phase, and the cell concentration was adjusted to 2 × 10^5^ cells/mL. The cells were seeded in 6-well culture plates according to the grouping, and each well of 6-well plates was 1 mL. Transfection was performed according to the grouping.

The transfection mixture was prepared according to the ratio of 125 μL basal medium 100 pmol, 4 μL Lipo3000, mixed gently, and incubated at room temperature for 20 min. After that, 5 μL transfection mixture was uniformly added to each well, and the mixture was incubated for 48 h at 37 °C in a 5% CO_2_ incubator.

The prepared mixed suspension was added according to the grouping and cultured in a constant temperature incubator at 37 °C for 4 to 6 h, and the cell state was observed. After another 48 h of culture, the samples were collected.

### 4.10. Reverse Transcription-Polymerase Chain Reaction (RT-PCR)

The concentration of cells in each group was adjusted to 1 × 10^5^ mL^−1^ and seeded in 6-well plates, with 2 mL in each well. The cells were cultured in the incubator for 24 h. After trypsin digestion, the cells were centrifuged at 2000 r/min for 10 min (r = 10 cm), and the total RNA was extracted by Trizol extraction kit (Invitrogen, Waltham, MA, USA).

The extracted total RNA was reverse-transcribed into cDNA for fluorescence quantitative PCR reaction according to the reverse transcription kit instructions. The primers were designed and synthesized by Shanghai Sangon Primer Premier 5.0 software, and the housekeeping gene Gapdh was used as an internal control. The 2^−ΔΔCT^ method was used to analyze the expression difference in target genes between the control group and each experimental group.

### 4.11. Western Blot Analysis

RIPA (Solarbio, Beijing, China) lysate can be used to lyse cells, and the extract can obtain the supernatant. Next, the protein concentration in the supernatant was measured using a BCA protein assay kit (Biosharp, Hefei, China). The protein sample was then denatured by adding the loading buffer at 100 °C for 5 min (Beyotime, Haimen, China). The proteins were loaded onto a 10% SDS-PAGE gel and transferred to a PVDF membrane (GE, Boston, MA, USA). The membrane was subsequently blocked with 5% skim milk for 1 h and incubated with the corresponding primary antibody (Abcam, Cambridge, UK) at 4 °C for 24 h. Next, it was incubated with the HRP-conjugated secondary antibody (Transgon, Beijing, China) for 1 h at room temperature. Finally, we used a hypersensitive chemiluminescence kit to develop the protein bands.

### 4.12. CCK-8 Assay

Cell counting kit-8 (Shanghai Yunbelt Technology, Ltd., Shanghai, China) was used to evaluated cell viability. In a 96-well plate, we seeded the cells (1500 cells/well). CCK-8 reagent was then added (20 μL/well) after culturing the cells to different time points (0 h, 12 h, 24 h, 36 h, 48 h, 60 h, 72 h, 84 h, 96 h). Next, the 96-well plate was incubated in the cell incubator at 36 °C for 2 h. The number of live cells in the culture was determined by measuring the absorbance at 450 nm after incubation.

### 4.13. Cell Wound Healing Experiment

Cells were seeded in 6-well plates at a density of 5 × 10^5^ cells/well. At approximately 90% confluence, the well plate was nicked vertically with the tip of a 200 µL single-pass pipette gun. The shed cells were then washed off with PBS, and serum-free medium was used for further culture. After siRNA NC and siRNA CDC6 transfection, the width of the scratch was observed and statistical analysis was performed for each time point (0 h and 48 h).

### 4.14. Transwell Experiment

Matrigel originally stored at −20 °C was thawed at 4 °C overnight. It was subsequently diluted in serum-free DMEM to a volume ratio of 3:1. The prepared artificial basement membrane (100 μL) was added to the upper chamber and incubated at 37 °C for 2–6 h to reach a gel-like consistency. After transfection for 48 h, siRNA NC and siRNA CDC6 transfected cells were collected in each group, and the cell concentration was adjusted. Serum-free cell suspension (300 μL) was added to the upper chamber, whereas 600 μL serum-containing medium was added to the lower chamber. The chamber and the liquid from the upper chamber were removed after 48 h of culture. The non-invasive cells and artificial basement membrane glue on the membrane were then carefully wiped with a cotton swab. Next, they were rinsed twice with PBS pre-warmed at 37 °C, fixed with 4% paraformaldehyde pre-cooled in ice for 30 min, and stained with crystal violet for 10 min. The polycarboester membrane was subsequently cut from the base of the upper chamber. The cells infiltrating the back of the chamber were counted under a microscope after sealing the piece, and photographs were taken with a light microscope in random fields.

### 4.15. Flow Cytometry

After transfection for 48 h, siRNA NC and siRNA CDC6 transfected cells were collected in each group, and the cell concentration was adjusted. Cells were washed with PBS to reach single-cell suspensions in 100 µL of PBS. They were then stained with 5 µL of Annexin V/PI apoptosis detection kit and 250 µL buffer (Apexbio, Shanghai, China) for 30 min in the dark according to the manufacturer’s instructions.

### 4.16. Statistical Analysis

Statistical analyses were performed using R statistical package (R Foundation for Statistical Computing, Vienna, Austria) or GraphPad Prism 8 (GraphPad Software, Boston, MA, USA). In addition, statistical differences between the experimental and control groups were determined using Student’s *t*-test. *p* < 0.05 was considered statistically significant.

## Data Availability

The original cohort data presented in the study are included in the Supplementary Tables. More information is available upon reasonable request from the corresponding author.

## Supplementary Materials

The following supporting information can be downloaded at https://www.mdpi.com/article/10.3390/ijms252312974/s1.
