# Supplementary material for: Exploring Bioinformatics Tools to Analyze the Role of CDC6 in the Progression of Polycystic Ovary Syndrome to Endometrial Cancer by Promoting Immune Infiltration
Source: Int J Mol Sci. 2024 Dec 3;25(23):12974. doi: 10.3390/ijms252312974 (PMC11640967; doi:10.3390/ijms252312974)
Supplement: Supplementary file 1 [file ijms-25-12974-s001.zip › Supplementary Table 1.pdf]

Supplementary Table 1. In the dataset of PCOS samples with a tendency to cancer, the associated ssGSEA scores of the three immune cell types

	B cell	Innate immunity	Myeloid derived suppressor cell	T cell
GSM1174423	0.286111557	0.085507505	-0.220748143	0.197174838
GSM1174424	0.330124619	0.270123614	0.204412076	0.372634427
GSM1174425	0.273444709	0.28456426	0.564972331	0.286366875
GSM1174426	0.174900335	0.065586924	0.078671159	0.067004651
GSM1174427	0.208210202	0.128096408	0.112094812	0.205798404
GSM1174428	0.261994949	0.249423019	0.468234522	0.219441976
GSM1174429	0.402807234	0.28639409	0.692215342	0.249751582
GSM1174430	0.210496419	0.181619399	-0.079637054	0.131190478
GSM1174431	0.102267604	0.073056535	-0.307784658	0.061466956
GSM1174432	0.27556543	0.142701651	0.166591224	0.142648254
GSM1174433	0.049440223	0.190491717	0.237276629	0.23719665
GSM1174434	0.217157486	0.170240264	0.089846926	0.12892641
GSM1174435	0.148594777	0.151700438	0.075913784	0.083548426
GSM1174436	0.213757501	0.335388701	0.547436381	0.307001124

CC	ribosome	1.03E-09	1.83E-07	19
CC	large ribosomal subunit	8.13E-07	9.64E-05	11
CC	chromosomal region	1.11E-06	9.88E-05	20
CC	cytosolic ribosome	1.86E-06	0.000132573	10
CC	spindle pole	7.50E-06	0.000444614	12
CC	cytosolic large ribosomal subunit	1.95E-05	0.000993151	7
CC	methylosome	2.75E-05	0.001190466	4
CC	spliceosomal complex	3.10E-05	0.001190466	12
CC	mitochondrial protein-containing complex	3.35E-05	0.001190466	15
MF	structural constituent of ribosome	3.53E-11	1.60E-08	19
MF	protein transmembrane transporter activity	3.46E-05	0.007842362	5
MF	cadherin binding	6.34E-05	0.009580358	16
MF	macromolecule transmembrane transporter activity	0.000102511	0.011626901	5
MF	ubiquitin-like protein ligase binding	0.000140664	0.012763373	15
MF	protein transporter activity	0.000243875	0.018440356	5
MF	heat shock protein binding	0.000834451	0.04186949	8
MF	ubiquitin protein ligase binding	0.000835466	0.04186949	13
MF	GTPase binding	0.000914898	0.04186949	13
MF	protein tag	0.000922877	0.04186949	3
