# Supplementary material for: Exploring Bioinformatics Tools to Analyze the Role of CDC6 in the Progression of Polycystic Ovary Syndrome to Endometrial Cancer by Promoting Immune Infiltration
Source: Int J Mol Sci. 2024 Dec 3;25(23):12974. doi: 10.3390/ijms252312974 (PMC11640967; doi:10.3390/ijms252312974)
Supplement: Supplementary file 1 [file ijms-25-12974-s001.zip › Supplementary Table 2.pdf]

Supplementary Table 2. The associated ssGSEA scores of the three immune cell types in the endometrial cancer sample dataset

	B cell	Innate immunity	Myeloid derived suppressor cell	T cell
GSM3401519	0.2111444	0.5189407	0.8213501	0.5610048
GSM3401520	-0.1107265	0.3531049	0.2961661	0.3883584
GSM3401521	-0.02937278	0.34696771	0.05653196	0.35088566
GSM3401522	-0.1328141	0.3786337	0.2405013	0.3669955
GSM3401523	0.1329841	0.4642825	0.5131565	0.4915613
GSM3401524	0.0872339	0.4271604	0.24967	0.4023061
GSM3401525	-0.1250462	0.4205037	0.2968829	0.3903823
GSM3401526	-0.02856495	0.38130128	0.19773631	0.33318053
GSM3401527	-0.04832475	0.48975503	0.48586641	0.43898984
GSM3401528	-0.12293671	0.34232436	0.01690786	0.27064743
GSM3401529	0.08573689	0.44468543	0.386162	0.48085894
GSM3401530	0.1480363	0.5420099	0.7229624	0.5963968
GSM3401531	-0.07779702	0.34529806	0.04017061	0.34682579
GSM3401532	-0.03628887	0.38320254	0.26480342	0.33776534
GSM3401533	-0.1233171	0.3508498	0.1548046	0.3294878
GSM3401534	-0.09921118	0.38843693	0.25677749	0.3696078
GSM3401535	-0.1621748	0.39031369	0.07619797	0.28623495
GSM3401536	-0.03269778	0.38745679	0.19620627	0.31825433
GSM3401537	-0.07156702	0.43892432	0.35317218	0.42849363
GSM3401538	-0.05364069	0.40128217	0.45869845	0.42685337
GSM3401539	-0.06620317	0.36549043	-0.07396415	0.29667748
GSM3401540	0.1620148	0.4724187	0.5356728	0.4272673
GSM3401541	0.03069467	0.45715258	0.22842229	0.36261947
GSM3401542	-0.01512057	0.33266309	0.02821587	0.34781008
GSM3401543	-0.140051	0.4001676	0.1259347	0.3616643
GSM3401544	-0.04312316	0.43851993	0.43581051	0.42678404
GSM3401545	-0.07333291	0.43928855	0.30140205	0.42048925
GSM3401546	-0.1221534	0.3940574	0.3684374	0.386428
GSM3401547	-0.04963433	0.39638738	0.10935523	0.30229039
GSM3401548	-0.02457819	0.48037051	0.5581194	0.43443666
GSM3401549	-0.06550544	0.36761341	0.34751303	0.36899659
GSM3401550	0.05834395	0.44141928	0.33324989	0.42086767
GSM3401551	0.0875033	0.4410301	0.5118339	0.494167
GSM3401552	0.05768326	0.49766747	0.40732651	0.46912757
GSM3401553	-0.17864991	0.34659747	-0.04302906	0.27694134
GSM3401555	-0.05046555	0.39753305	-0.02879077	0.36001599
GSM3401559	-0.09853192	0.43206422	0.28881965	0.41933449
GSM3401563	0.05698531	0.46737712	0.42532925	0.48118686
GSM3401565	-0.158570237	0.363240029	0.003767484	0.391435381
GSM3401569	-0.04906986	0.4292262	0.21075065	0.42053242

GSM3401610	-0.02664452	0.40983337	0.11647887	0.34511341
GSM3401629	0.367918	0.5188002	0.5278434	0.5017076
GSM3401630	0.02689317	0.45940314	0.13994668	0.47843539
GSM3401631	-0.07021914	0.47975854	0.47466408	0.41382036
GSM3401632	-0.1676324	0.4863326	0.2991693	0.4266635
