# Supplementary material for: Exploring Bioinformatics Tools to Analyze the Role of CDC6 in the Progression of Polycystic Ovary Syndrome to Endometrial Cancer by Promoting Immune Infiltration
Source: Int J Mol Sci. 2024 Dec 3;25(23):12974. doi: 10.3390/ijms252312974 (PMC11640967; doi:10.3390/ijms252312974)
Supplement: Supplementary file 1 [file ijms-25-12974-s001.zip › Supplementary Table 3.pdf]

Supplementary Table 3. KEGG pathway analysis of significantly differentially regulated genes In PCOS samples with a tendency to become cancerous

ID	Description	*P* value	*Q* value	Count
hsa05205	Proteoglycans in cancer	8.82E-06	0.002496151	24
hsa04914	Progesterone-mediated oocyte maturation	0.000455755	0.051888749	13
hsa04114	Oocyte meiosis	0.000549751	0.051888749	15
hsa05231	Choline metabolism in cancer	0.001074209	0.076042725	12
hsa04360	Axon guidance	0.00241161	0.128781271	17
hsa05418	Fluid shear stress and atherosclerosis	0.002870129	0.128781271	14
hsa04110	Cell cycle	0.003249798	0.128781271	13
hsa01524	Platinum drug resistance	0.004225124	0.128781271	9
hsa00280	Valine, leucine and isoleucine degradation	0.004428391	0.128781271	7
hsa04068	FoxO signaling pathway	0.004548037	0.128781271	13
hsa04141	Protein processing in endoplasmic reticulum	0.00757526	0.188986914	15
hsa04150	mTOR signaling pathway	0.008009111	0.188986914	14
hsa04146	Peroxisome	0.009063132	0.197407496	9
hsa00640	Propanoate metabolism	0.0116598	0.224957434	5
hsa05218	Melanoma	0.012624814	0.224957434	8
hsa03022	Basal transcription factors	0.012711349	0.224957434	6
hsa00900	Terpenoid backbone biosynthesis	0.013912028	0.231723567	4
hsa04213	Longevity regulating pathway - multiple spe cies	0.01762587	0.260954653	7
hsa00520	Amino sugar and nucleotide sugar metabolis m	0.018883122	0.260954653	6
hsa04015	Rap1 signaling pathway	0.020914561	0.260954653	16
hsa04350	TGF-beta signaling pathway	0.02091644	0.260954653	9
hsa01250	Biosynthesis of nucleotide sugars	0.02115299	0.260954653	5
hsa01521	EGFR tyrosine kinase inhibitor resistance	0.021196502	0.260954653	8
hsa00230	Purine metabolism	0.023642673	0.278942062	11
hsa00563	Glycosylphosphatidylinositol (GPI)-anchor biosynthesis	0.024812127	0.28102999	4
hsa03460	Fanconi anemia pathway	0.029129189	0.306621211	6
hsa04510	Focal adhesion	0.029237301	0.306621211	15

hsa04152	AMPK signaling pathway	0.036211554	0.339897898	10
hsa01212	Fatty acid metabolism	0.036753681	0.339897898	6
hsa00071	Fatty acid degradation	0.037849515	0.339897898	5
