# Supplementary material for: Exploring Bioinformatics Tools to Analyze the Role of CDC6 in the Progression of Polycystic Ovary Syndrome to Endometrial Cancer by Promoting Immune Infiltration
Source: Int J Mol Sci. 2024 Dec 3;25(23):12974. doi: 10.3390/ijms252312974 (PMC11640967; doi:10.3390/ijms252312974)
Supplement: Supplementary file 1 [file ijms-25-12974-s001.zip › Supplementary Table 4.pdf]

Supplementary Table 4. GO enrichment analysis of three important components of up-regulated significantly differential genes In PCOS samples with a tendency to become cancerous

Ontology	Description	*P* value	Q value	Count
BP	cilium assembly	5.85E-10	2.24E-06	45
BP	cilium organization	9.97E-10	2.24E-06	46
BP	nuclear division	8.39E-08	0.000115297	44
BP	ciliary basal body-plasma membrane doc king	1.03E-07	0.000115297	18
BP	regulation of mitotic nuclear division	1.31E-07	0.00011802	24
BP	smoothened signaling pathway	1.75E-07	0.000131347	22
BP	microtubule cytoskeleton organization in volved in mitosis	2.25E-07	0.000144788	22
BP	mitotic nuclear division	2.59E-07	0.00014566	33
BP	centrosome cycle	3.70E-07	0.000185144	20
BP	regulation of nuclear division	5.29E-07	0.000238026	25
CC	ciliary basal body	9.54E-11	4.98E-08	28
CC	spindle	1.86E-07	4.86E-05	39
CC	ciliary tip	3.63E-07	6.32E-05	12
CC	centriole	3.17E-06	0.000413714	20
CC	kinetochore	6.30E-06	0.00065745	19
CC	spindle pole	8.26E-06	0.00071878	21
CC	intraciliary transport particle	1.19E-05	0.000887128	8
CC	condensed chromosome outer kinetochor e	1.37E-05	0.000892013	6
CC	condensed chromosome	3.14E-05	0.001822502	24
CC	chromosome, centromeric region	3.74E-05	0.00195514	22

MF	tubulin binding	2.46E-06	0.002052913	37
