# Supplementary material for: Exploring Bioinformatics Tools to Analyze the Role of CDC6 in the Progression of Polycystic Ovary Syndrome to Endometrial Cancer by Promoting Immune Infiltration
Source: Int J Mol Sci. 2024 Dec 3;25(23):12974. doi: 10.3390/ijms252312974 (PMC11640967; doi:10.3390/ijms252312974)
Supplement: Supplementary file 1 [file ijms-25-12974-s001.zip › Supplementary Table 5.pdf]

Supplementary Table 5. GO enrichment analysis of three important components of down-regulated significantly differential genes in the endometrial cancer sample dataset

Ontology	Description	*P* value	Q value	Count
BP	negative regulation of cell cycle phase transition	1.64E-10	3.57E-07	22
BP	mitotic cell cycle checkpoint signaling	3.24E-10	3.57E-07	16
BP	chromosome segregation	4.48E-10	3.57E-07	27
BP	cell cycle checkpoint signaling	4.71E-10	3.57E-07	18
BP	negative regulation of mitotic cell cycle	7.42E-10	3.97E-07	20
BP	negative regulation of mitotic cell cycle phase transition	7.87E-10	3.97E-07	18
BP	negative regulation of cell cycle process	2.58E-09	1.12E-06	22
BP	nuclear chromosome segregation	8.84E-09	3.35E-06	22
BP	negative regulation of cell cycle	1.03E-08	3.46E-06	24
BP	nuclear division	7.94E-08	2.41E-05	24
CC	ribosomal subunit	2.16E-10	7.68E-08	18
