# Supplementary material for: Exploring Bioinformatics Tools to Analyze the Role of CDC6 in the Progression of Polycystic Ovary Syndrome to Endometrial Cancer by Promoting Immune Infiltration
Source: Int J Mol Sci. 2024 Dec 3;25(23):12974. doi: 10.3390/ijms252312974 (PMC11640967; doi:10.3390/ijms252312974)
Supplement: Supplementary file 1 [file ijms-25-12974-s001.zip › Supplementary Table 6.pdf]

**Supplementary Table 6.** KEGG pathway analysis of significantly differentially regulated genes in the endometrial cancer sample dataset

| ID | Description | *P* value | Q value | Count |
|---|---|---|---|---|
| hsa03010 | Ribosome | 2.71E-07 | 5.63E-05 | 15 |
| hsa03013 | Nucleocytoplasmic transport | 0.000130977 | 0.013580213 | 9 |
| hsa03015 | mRNA surveillance pathway | 0.000334501 | 0.023121652 | 8 |
| hsa05171 | Coronavirus disease - COVID-19 | 0.00092355 | 0.047878771 | 12 |
| hsa05110 | Vibrio cholerae infection | 0.00192333 | 0.069556301 | 5 |
| hsa04110 | Cell cycle | 0.002012543 | 0.069556301 | 9 |
| hsa00190 | Oxidative phosphorylation | 0.002763899 | 0.081877905 | 8 |
| hsa05418 | Fluid shear stress and atherosclerosis | 0.003464447 | 0.089802111 | 8 |
| hsa05014 | Amyotrophic lateral sclerosis | 0.00568357 | 0.123097065 | 14 |
| hsa04145 | Phagosome | 0.005936153 | 0.123097065 | 8 |
| hsa04114 | Oocyte meiosis | 0.009117823 | 0.17188624 | 7 |
| hsa04210 | Apoptosis | 0.011076533 | 0.191410256 | 7 |
| hsa04721 | Synaptic vesicle cycle | 0.012854113 | 0.204567916 | 5 |
| hsa04120 | Ubiquitin mediated proteolysis | 0.01381093 | 0.204567916 | 7 |
| hsa00020 | Citrate cycle (TCA cycle) | 0.016117218 | 0.222813468 | 3 |
| hsa05016 | Huntington disease | 0.02158085 | 0.279699177 | 11 |
| hsa04141 | Protein processing in endoplasmic reticulum | 0.03312102 | 0.374884693 | 7 |
| hsa05211 | Renal cell carcinoma | 0.03502146 | 0.374884693 | 4 |
| hsa05165 | Human papillomavirus infection | 0.035560803 | 0.374884693 | 11 |
| hsa04914 | Progesterone-mediated oocyte maturation | 0.036156392 | 0.374884693 | 5 |
| hsa03040 | Spliceosome | 0.040371082 | 0.387867061 | 8 |
| hsa01524 | Platinum drug resistance | 0.04176692 | 0.387867061 | 4 |
| hsa05152 | Tuberculosis | 0.043019773 | 0.387867061 | 7 |
| hsa05166 | Human T-cell leukemia virus 1 infection | 0.046179708 | 0.399008877 | 8 |

| | | | | |
|---|---|---|---|---|
| hsa05412 | Arrhythmogenic right ventricular cardiomyopathy | 0.049197683 | 0.408081835 | 4 |
