# Supplementary material for: Exploring Bioinformatics Tools to Analyze the Role of CDC6 in the Progression of Polycystic Ovary Syndrome to Endometrial Cancer by Promoting Immune Infiltration
Source: Int J Mol Sci. 2024 Dec 3;25(23):12974. doi: 10.3390/ijms252312974 (PMC11640967; doi:10.3390/ijms252312974)
Supplement: Supplementary file 1 [file ijms-25-12974-s001.zip › Supplementary Table 7.pdf]

Supplementary Table 7. In the dataset of PCOS samples with a tendency to become cancerous, LASSO regression analysis evaluates the correlation between core genes and biomarkers

**(Intercept)**	**2.9080373**
HSPB11	-
AP2S1	6.0349146
BMPR2	1.1218302
HAUS6	-
EXOSC3	1.5729001
CDC6	1.0465043
EGFR	-

Supplementary Table 8 | In the endometrial cancer sample dataset, LASSO regression analysis evaluates the correlation between core genes and biomarkers

**(Intercept)**	**-9.8058588**
HSPB11	0.6859017
AP2S1	-
BMPR2	-
HAUS6	-0.2366846
EXOSC3	0.1064983
CDC6	0.3578251
EGFR	-0.3344211
